# A Survey of Research on Vibration Friction Reduction Technologies in Aero-Engines

**DOI:** 10.3390/ma18030535

**Published:** 2025-01-24

**Authors:** Yunnan Teng, Jingyang Ma, Liyang Xie

**Affiliations:** Department of Mechanical Engineering and Automation, Northeastern University, Shenyang 110819, China; wzwz7892022@126.com (J.M.); xielyneu@126.com (L.X.)

**Keywords:** aero-engine, vibrational friction, frictional force, overview, 74H45

## Abstract

The performance and reliability of an aviation engine, as a core component of an aircraft, is of paramount importance to flight safety. Scholars worldwide have contributed to the design and research of aviation engines, and these advancements and contributions embody the relentless efforts and innovative spirit of scholars and engineers in the field of aviation engine design research. In recent studies, researchers have proposed various vibration control methods to address friction issues in aviation engines, offering corresponding control strategies for the aspects of materials, lubrication, and structural design. However, how to reduce the loss of service life and the safety risks caused by friction in aircraft engines has become an urgent issue that needs to be addressed in the aviation sector. This paper briefly analyzes the current development status regarding vibration and friction reduction in aircraft engines, explores key technologies and research progress in addressing this challenge, and provides insights and prospects for future developments. With advancements in technology, research into vibration and friction in aviation engines will continue to deepen, providing strong support for the development of the aviation industry.

## 1. Introduction

In the early 20th century, the success of aircraft design established aerodynamics as a key area of research, with friction drag identified as a critical factor affecting flight performance. Several outstanding experts and scholars have contributed valuable research to the field. Ludwig Prandtl introduced the boundary layer theory, significantly advancing the understanding of frictional forces and laying the foundation for subsequent research on aerodynamic friction [1]. Building on Prandtl’s work, Theodore von Kármán delved deeply into fluid dynamics and aerodynamic friction drag, covering critical areas such as turbulence and boundary layer theory [2].

After World War II, J.A. Fay utilized equilibrium and non-equilibrium numerical methods to focus on surface friction in supersonic flows. His research revealed that thermal transfer in dissociated air could be accurately calculated through the equilibrium stagnation boundary layer. These findings provided a theoretical basis for the design of hypersonic vehicles and the optimization of thermal protection systems [3]. Around the same period, the US began focusing on vibration issues, though efforts were largely limited to basic vibration analysis and reducing vibration transmission.

As aerodynamic theories advanced, J.D. Anderson, in 1991, extensively discussed the issue of friction in aerodynamics. He summarized the fundamental principles of aerodynamics, including the basic properties of fluids, fluid statics, fluid dynamics, flow analysis, and the control volume method. His work also provided performance parameters for isentropic flows and normal shock waves while proposing optimized engine structures [4]. During the same period, Japan began employing modern sensors to identify vibration sources in engines, while Russia prioritized vibration analysis under extreme temperature and pressure conditions [5]. Furthermore, the widespread application of modern digital simulation technologies enriched methods for addressing aviation friction issues. Researchers such as Piotr Wróblewski and Robert Rogólski investigated the impact of variations in the tribological parameters of coatings like TiN, TiAlN, CrN, and DLC1 on the resistance torque of piston motion in aircraft engines. Their studies, which simulated real engine operating conditions, found that the coatings on piston ring sliding surfaces and cylinder running surfaces were decisive in determining changes in engine motion resistance torque [6].

In practical applications, global companies such as Boeing, Airbus, Rolls-Royce, Honeywell International, and Northrop Grumman have developed specialized materials and technologies to reduce friction in various types of engines. Rolls-Royce, founded in 1906 and one of the world’s leading aircraft engine manufacturers, focuses on reducing friction losses through engine design to improve flight efficiency. Its friction-reducing blade technology minimizes air resistance through engine fan blade designs that optimize airflow paths. Honeywell International has optimized turbine engines using advanced combustion and airflow management technologies, reducing friction losses while improving fuel efficiency. Its HTF7000 series turbine engines power a wide range of commercial and business aircraft.

From the 1980s to the 1990s, as the performance requirements of aircraft engines increased, research in China shifted toward material science and coating technologies for friction and vibration reduction. High-temperature alloys, ceramic coatings, and other advanced materials were widely studied and gradually applied to critical components of aircraft engines. These developments significantly promoted the use of high-performance aviation materials, particularly those suited for high-temperature and high-pressure conditions, such as high-temperature alloys and ceramic coatings, which greatly enhanced the efficiency and service life of aircraft engines. In vibration reduction, the Zhuzhou Aero-Engine Research Institute developed a “gas counter-swirl” active control system for rotating machinery, which suppresses transverse rotor vibrations caused by imbalance factors by increasing effective system damping.

In the 21st century, aviation friction and vibration reduction technologies entered a new phase, with research focusing on composite materials, self-lubricating materials, and intelligent lubrication systems. Composite materials enable aircraft engines to maintain high efficiency under high-temperature and high-speed conditions. Intelligent lubrication systems, which emerged during this period, utilize sensors and feedback mechanisms to dynamically adjust lubricant supply based on real-time operating conditions, further improving engine efficiency and safety.

Since 2010, with continuous technological advancements, aerospace vibration and friction reduction materials and lubrication systems have entered a new stage of intelligence and refinement. Nanotechnology and intelligent lubrication systems have become research hotspots in domestic and foreign aerospace vibration and friction reduction technologies. Research during this period indicates that with the development of nanotechnology and intelligent lubrication systems, aerospace friction reduction technology will enter a new era, significantly enhancing the performance and reliability of aircraft.

In the field of practical product applications, China has established numerous innovative aviation enterprises, such as the Aviation Industry Corporation of China (AVIC) and Shenyang Aircraft Corporation (SAC), which have made significant contributions to the research and application of friction reduction technologies. AVIC, founded in 1951, is China’s largest aerospace manufacturing enterprise, covering multiple areas including commercial aircraft, military aircraft, and helicopters. In the field of aviation friction reduction, AVIC has successfully incorporated advanced composite materials, such as carbon fiber composites, into aircraft manufacturing to reduce weight, lower aerodynamic drag during flight, and improve fuel efficiency. For instance, over 20% of the domestically produced C919 large passenger aircraft consists of composite materials. Additionally, AVIC optimized aerodynamic designs through computational fluid dynamics (CFD) technology, improving the aerodynamic efficiency of the fuselage and wings to reduce in-flight friction losses and enhance cruising efficiency. The Aero-engine Corporation of China (AECC), which was established in 2016, focuses on the development and manufacturing of aircraft engines, promoting the localization of aviation engines in China. In the field of friction reduction, AECC has achieved advanced turbine blade designs. By optimizing the aerodynamic design of turbine blades, it has reduced air friction losses, enhancing engine efficiency. These advanced designs significantly reduce drag and improve the fuel economy of engines. H. U. Minghui systematically reviewed and analyzed the existing vibration fault diagnosis technologies for aero-engines, both domestically and internationally, from three aspects, and specifically included related technologies such as dynamic analysis, signal processing, and deep learning [7]. R. Holmes studied a test facility, which had the purpose of studying the phenomena experienced in an actual engine, which relate to system nonlinearities [8]. W. Xin summarized the types and mechanisms of machining deformation, and the machining deformation control of thin-walled casings. A comprehensive review on the types of machining deformation and machining deformation control methods of aero-engine thin-walled casings were discussed [9]. K. S. Hodgkinson studied gas turbine aero-engine rotor balance and vibration [10]. R. Yu proposed valuable suggestions of smart structure aero-engine blades and some vibration suppression strategies for aero-engine blades using piezoelectric materials were summarized [11]. Y. Zhang summarized the mathematical model of the blade and the deficiencies and disadvantages in the current research on blade modeling [12]. Luca Pugi proposed an innovative planar piezoelectric actuation stage. A design procedure was presented to apply to a benchmark test case and excitations were applied to study the characteristics of bearings of rotating machines [13,14].

In summary, vibration and friction reduction technologies in aerospace engines play a crucial role in the modern aviation industry, directly affecting engine reliability, efficiency, and lifespan. However, the current literature on aerospace engine vibration control lacks a comprehensive review of vibration and friction reduction technologies for aero-engines. Therefore, the contribution of this manuscript is to provide a comprehensive and in-depth overview that considers vibration and friction reduction through key technologies, the vibration friction model, and the application of vibration friction technology in the field of aero-engines. It explores key technologies and research progress in tackling this challenge and offers insights and prospects for future developments. With the application of new technology, advancements in materials science, nanotechnology, and intelligent manufacturing, vibration and friction reduction systems will strongly support the development of the aviation industry and its sustainable growth.

## 2. Key Technologies for Vibration and Friction in Aero-Engines

In practical mechanical engineering, it is necessary to simultaneously reduce internal friction in engines across various aspects and based on different criteria. Regarding aerospace engine materials, according to the “Preface to the Column on Surface Coatings for Aero-Engine Applications and Their High-Temperature Tribological Properties”, the document introduces various design and analysis methods for high-temperature composite coatings, preparation methods for multiple novel high-temperature resistant coatings, their microstructures, and their tribological properties, and corrosion resistance at high temperatures, as well as research findings on the impact of surface textures on fretting wear. This provides a wealth of references for researchers in surface engineering for aerospace engines and related energy and power equipment, as well as for scientists working in fields such as high-temperature tribology, corrosion, and lubrication [15]. In terms of structure, due to the complexity of aero-engine structures, this section only covers one aspect: The “Scraping Damage and Prevention Measures of Aero-Engine Bearings” points out the mechanism and hazards of scraping damage in the main bearings of aero gas turbine engines, and based on the mechanism of scraping damage, proposes two measures to prevent scraping damage in bearings: increasing the dragging force that drives roller–cage motion, and reducing the resistance that hinders roller–cage motion [16].

### 2.1. Main Design Requirements and Characteristics

Aircraft engine vibration and friction reduction systems have critical design and operational requirements aimed at reducing energy loss, heat buildup, and wear caused by friction, ensuring the efficient and reliable operation of engines. The key functional requirements are as follows.

#### 2.1.1. Material and Lubricant Requirements

Materials should have excellent high-temperature performance, corrosion resistance, strength, stiffness, low weight, ease of processing, and cost-effectiveness. Lubricants should possess high-temperature oxidation resistance, low viscosity, corrosion resistance, low volatility, and low toxicity to ensure stable engine operation under extreme conditions, extend service life, and improve overall performance and safety [17].

#### 2.1.2. Heat Dissipation Requirements

During operation, engines require a continuous supply of clean lubricant to bearings and transmission gears to reduce wear and dissipate heat generated by vibration and friction [18]. The system must be designed to effectively remove frictional heat, maintain the system temperature within a safe range, and ensure normal operation under extreme conditions.

#### 2.1.3. Transmission System Integration

The design of the vibration and friction reduction system must work closely with the transmission system, which includes gears and bearings, to ensure proper load distribution and avoid localized overload and wear. For example:

(1) Gear Transmission System: Gear meshing surfaces are friction hotspots. Their design must ensure optimized tooth profiles, gear clearances, and lubrication pathways to minimize friction and heat generation.

(2) Bearing System: Different types of bearings (e.g., ball bearings, sliding bearings) must closely interact with surrounding mechanisms, with appropriate installation methods and lubrication methods selected to accommodate high-speed rotation and reduce friction. Under high loads, the outer rings and rolling elements of rolling bearings may experience severe spalling, with the primary modes of bearing damage being contact fatigue spalling and abrasive wear. Contact fatigue damage in bearings occurs due to the accumulation of plastic strain and the formation of white, high-hardness “butterfly” structures under high loads [19]. When combining sliding bearings and rolling bearings, load distribution between them under different operating conditions must also be considered.

The application of vibration reduction systems in aerospace engines manifests across multiple critical components. The combined use of sleeve bearings and rolling bearings, through optimized lubrication channel design and material selection, effectively reduces friction and wear in rotating parts, particularly ensuring extended bearing lifespan at high speeds. Advanced high-temperature resistant coatings, such as ceramic coatings, are often employed at turbine blade roots to mitigate friction arising from vibrations between the blades and hub, thereby preventing material deformation and wear caused by elevated temperatures. Furthermore, in gear transmission systems, the adoption of synthetic lubricants and precise tooth profile designs significantly diminishes frictional losses between gears, enhancing transmission efficiency. By utilizing gas film seals and floating seal technology, the sealing system achieves low-friction operation between rotors and stators, maintaining sealing performance under high-temperature and high-pressure conditions to prevent lubricant leakage. These practical applications ensure the reliability and performance of the friction-reducing system under high-speed and high-temperature operating conditions of the engine, substantially enhancing the efficiency and safety of aerospace engines.

### 2.2. Design and Key Technology of Vibration Friction System

By clarifying the above requirements and characteristics, the main technologies of the friction reduction system at the engineering application level can be summarized.

(1) Application of anti-friction materials and coatings in aero-engines. Aero-engines have strict requirements for high-temperature materials and high-temperature resistant coating materials. For high-temperature materials, the application of ceramic matrix composites, carbon/carbon composites, and intermetallic compounds is mainly focused on at home and abroad [18]. This section only examines ceramic matrix composites in detail. Ceramic matrix composites have the characteristics of low density, high temperature resistance, oxidation resistance, etc., and their working temperature can be reached; compared with high-temperature alloy materials, they have a lower density, stronger oxidation resistance and high temperature resistance. Ceramic matrix composites can also be combined with silicon carbide fibers to form 1650 °C silicon carbide matrix composites (composites) SiC−SiC [20]. Compared to superalloys, the CMC−SiC cooling gas flow can be reduced and the 15–25% operating temperature can be increased without air cooling and thermal barrier coating at 150–350 °C, with a potential service temperature of up to 1650 °C, while achieving weight reduction. Compared to ceramic materials, it improves brittleness and defect susceptibility and suppresses defect volume effects, improving reliability. Compared to composite materials, it can improve oxidation resistance, strength, and service life. It is one of the key thermal structural materials with the most potential for high-temperature parts of aero-engines with a high thrust-to-weight ratio [21]. Among them, the material of the ceramic layer of the thermal barrier coating must have important physical and chemical properties, such as refractory qualities, phase stability, chemical inertness, low thermal conductivity, and high thermal reflectivity, and the correspondence between the thermal expansion coefficient and the base material must be considered. Now it is widely used, it has the excellent comprehensive characteristics of thermal barrier coating materials, with a high melting point, chemical stability, and high temperature oxidation, etc., but a pure allotropic deformation, after the temperature rises, from a monostatic structure to a cubic structure, accompanied by a change in volume fraction with the internal thermal stress as a stabilizer in order to alleviate the thermal expansion coefficient mismatch between the intermediate bonding layer and the ceramic layer due to the internal thermal stress caused by free expansion and contraction to achieve the hardening effect. This improves thermal shock resistance and the material’s lifetime.
(2) Lubricating oil serves as the “mechanical power transmission medium” in aircraft engine systems, performing crucial and multifaceted roles for the friction components of the engine. These roles include providing the necessary lubrication to reduce friction and wear, implementing effective thermal conduction for cooling, removing impurities generated by wear to maintain system cleanliness, ensuring sealing to prevent leaks, and preventing rust and corrosion by forming protective films. Consequently, it is widely used across various engine friction interfaces. Based on the SAE ASS5780D performance specifications, RIPP4058 aviation turbine engine lubricating oil has been developed both domestically and internationally. Research has shown that 4058 lubricating oil exhibits a more stable acid value and viscosity at high temperatures, superior resistance to high-temperature coking, and excellent material compatibility [22]. The specific properties are detailed in the following Table 1 and Table 2:
(3) In aerospace engines, bearings are required to endure not only complex dynamic loads but also extreme operating conditions, such as high temperatures, high speeds, and high-pressure environments. To minimize friction and enhance the operational lifespan and reliability of bearings, the selection of bearing type and structural optimization represent crucial steps in the design process. Among them, airfoil bearings, which utilize air as the lubricating medium, hold significant promise.
(4) The structural design of airfoil bearings facilitates the formation of a stable air film between the rotating and stationary components, thereby eliminating solid-to-solid contact friction. Their primary advantage lies in their extremely low friction coefficient, making them particularly suitable for high-speed applications and environments requiring oil-free lubrication. Currently, simulations of airfoil bearings have been conducted using COMSOL, and dynamic stiffness analysis has been completed [23].

## 3. Model

The aero-engine vibration friction model is a model that describes the friction force and its influence caused by contact and relative motion between blades, wheel disks, and other components of an aero-engine during vibration. This friction not only affects the operational stability and safety of aero-engines, but can also lead to wear and damage to components. It can be verified by physical model construction, mathematical simulation, and experiments, and must follow the three principles of accuracy, conciseness, and interpretability, and finally ensure its reliability by comparing the actual results with the simulation results.

The efficient and stable operation of the aero-engine depends on the synergistic effect of the rotor, stator, casing, and rolling bearing. The rotor is the core component that converts energy to drive flight, and the stator holds the support and directs the airflow, optimizing the aerodynamic efficiency. As an important supporting and load-bearing component, the casing ensures that the engine structure is stable and can withstand thrust. The rolling bearing support transmits the rotational force of the rotor to ensure stable positioning and withstand extreme environmental shocks. Together, they ensure the reliable operation and flight safety of aero-engines under extreme conditions.

In terms of the vibration of the whole machine, the rotation of the rotor of the core machine is particularly critical. Therefore, the synchronous circular motion model of the rotor is established, and the radial vibration model of the rotor is derived according to the cosine theorem and the geometric relationship:

(1) $$D=D_{0}+\left(R-r\right)-\left(R-r\right)\cdot \cos\left(\alpha \right)$$

where

R—the radial radius of the inscribed circle,

r—the radius of the rotor circler,

D—clearance value,

$D_{0}$—the DC component.

By studying the motion characteristics of the rotor during the working process, the rotor vibration and elastic line changes in the process of vibration abrupt change can be revealed, and the dynamic mechanism in the process of vibration abrupt change can be explored from the perspective of rotor motion, to further confirm the cause of the vibration characteristics of the whole machine [24].

In the aero-engine machine, the stator casing has the function of bearing a variety of loads, supporting the stator structure, affecting the performance of the compressor and the dynamic characteristics of the whole machine. Through the analysis of the intrinsic characteristics of the stator casing in the free mode, it is inferred that

(2) $$\omega^{2}I\varphi =M^{-1}K\varphi \omega$$

where

I—identity matrix,

φ—the mode shape,

M—mass matrix,

K—the stiffness matrix are obtained.

At the same time, it is found that when the bolt preload is applied to the whole machine casing, the overall connection structure will produce an additional stiffness ΔK, which can be obtained by combining with the previous model:

(3) $$\Delta \omega^{2}I\varphi =M^{-1}\Delta K\varphi \frac{\Delta \omega^{2}}{\omega^{2}}=\frac{∥M^{-1}\Delta K\varphi ∥}{∥M^{-1}K\varphi ∥}$$

where

$\Delta \omega^{2}$—variation in the natural frequency caused by the application of the bolt preload.

$\Delta \omega^{2}$ can be obtained in relation to the mass matrix, the structural stiffness, and the resulting additional stiffness when the preload is applied. The influence of bolt preload on the vibration response of the whole casing, the uniform distribution of bolt preload, and the non-uniform distribution of bolt preload are effectively explained [25].

In terms of dynamic modeling, there are support connections between the rotor and the casing in the whole machine, respectively (for the differential equation of motion of the support between the rotor and the casing in each section $RC_{i}$, the differential equation of motion is that

(4) $$\left\{\begin{array}{l} m_{bi}{\ddot{x}}_{bi}+k_{ti}(x_{bi}-x_{wi})-F_{dxi}=F_{xci}\\ m_{bi}{\ddot{y}}_{bi}+k_{ti}(y_{bi}-y_{wi})-F_{dyi}=F_{yci}-m_{bi}g \end{array}\right.\;i=1,\;2,\cdots N.$$

where

The i-th support of RC is connected to the m-th node of the rotor and the n-th node of the casing,

$m_{bi}$—mass of the bearing seat,

$k_{ti}$—linear stiffness between the outer ring of the bearing and the bearing housing,

$x_{bi}$—the lateral displacements of the bearing housing at support $RC_{i}$,

$y_{bi}$—the vertical displacements of the bearing housing at support $RC_{i}$,

$x_{wi}$—the lateral displacement of the i-th node of the rolling bearing’s outer ring,

$y_{wi}$—the vertical displacement of the i-th node of the rolling bearing’s outer ring,

$F_{dxi}$, $F_{dyi}$—the damping forces,

$F_{xci}$, $F_{yci}$—the linear elastic forces acting on the bearing housing from the casing.

If the linear spring connection between rotor and casing (for the elastic connection between rotor and casing $RCC_{k}$ (k = 1, 2, …, N)), lets the ith node of the rotor relate to the jth node of the casing, then there is a model:

(5) $$\begin{array}{l} \left\{\begin{array}{l} F_{rxi}=k_{gr}(x_{cj}-x_{ri})+c_{gr}({\dot{x}}_{cj}-{\dot{x}}_{ri})\\ F_{ryi}=k_{gr}(y_{cj}-y_{ri})+c_{cr}({\dot{y}}_{cj}-{\dot{y}}_{ci})\\ M_{rxi}=k_{ga}(\phi_{cj}-\phi_{ri})+c_{ga}({\dot{\phi }}_{cj}-{\dot{\phi }}_{ri})\\ M_{ryi}=k_{ga}(\psi_{cj}-\psi_{ri})+c_{ga}({\dot{\psi }}_{cj}-{\dot{\psi }}_{ri}) \end{array}\right.\\ \left\{\begin{array}{l} F_{cxj}=-F_{rxi}\\ F_{cyj}=-F_{ryi}\\ M_{cxj}=-M_{rxi}\\ M_{cyj}=-M_{ryi} \end{array}\right. \end{array}$$

where

$k_{gr}$—the radial stiffness of the connection,

$k_{ga}$—the angular stiffness,

$c_{gr}$—the radial damping

$c_{ga}$—the angular damping

$x_{ri}$, $y_{ri}$, $\phi_{ri}$, $\psi_{ri}$—the displacements of the i-th node of the rotor,

$x_{cj}$, $y_{cj}$, $\phi_{cj}$, $\psi_{cj}$—the displacements of the j-th node of the casing,

$F_{rxi}$, $F_{ryi}$, $M_{rxi}$, $M_{ryi}$—the forces and moments acting on the i-th node of the rotor,

$F_{cxj}$, $F_{cyj}$, $M_{cxj}$, $M_{cyj}$—the forces and moments acting on the j-th node of the casing.

The coupling connection between the rotor and the casing foundation (installation section) elastic support (for the elastic supports $CB_{k}$ (k = 1, 2, …, N)) at the mounting joints between the casing and the foundation, assume that the ith node of the casing is connected to the foundation with elastic supports. There is a model [26,27]:

(6) $$\left\{\begin{array}{l} F_{cxi}=-k_{cx}x_{ci}-c_{cx}{\dot{x}}_{ci}\\ F_{cyi}=-k_{cy}y_{ci}-c_{cy}{\dot{y}}_{ci} \end{array}\right.$$

where

$k_{cx}$, $k_{cy}$—the connection stiffness in the support directions,

$c_{cx}$, $c_{cy}$—the connection damping,

$x_{ci}$, $y_{ci}$—the displacements of the i-th node of the casing,

$F_{cxi}$, $F_{cyi}$—the forces acting on the i-th node of the casing.

These fundamental models collectively constitute a discrete support model.

Although the current rolling bearing model is relatively perfect, the existing research work is based on the simple rotor model, and the rolling bearing model is not applied to the complex rotor support casing coupling dynamic model. The differential equation of motion for the outer ring of a rolling bearing is:

(7) $$Rr\omega_{Cage}=\frac{\omega \times r}{R+r}\omega \left\{\begin{array}{l} m_{wi}{\ddot{x}}_{wi}+k_{ti}(x_{wi}-x_{bi})+F_{dxi}=F_{xRi}\\ m_{wi}{\ddot{y}}_{wi}+k_{ti}(y_{wi}-y_{bi})+F_{dyi}=F_{yRi}-m_{wi}g \end{array}\right.\;i=1,\;2,\cdots N$$

where

$m_{w}$—the mass of the outer ring,

$x_{w}$—the displacement of the outer ring,

$k_{ti}$—the stiffness of the connection between the outer ring and the housing,

$F_{dxi},F_{dyi}$—the damping forces.

Although this method establishes an accurate vibration model of the whole machine, it is computationally expensive and complex, and consumes a lot of resources.

The vibration modeling analysis of the whole machine can accurately reflect the actual vibration behavior of the engine, but the overall model is more complex and more likely to be affected by other factors, which increases the instability, so local vibration analysis is more necessary.

Local vibration friction analysis: It is very important to study the local friction problem in aero-engines, especially the friction phenomenon of high-pressure turbine disks and low-pressure turbine disks under the constraints of the receiver, and to construct an accurate friction model of the single rotor–rolling bearing–receiver coupling system, which is very important for predicting the failure trend, formulating maintenance strategies, ensuring the stability and safety of the engine, improving reliability and durability, optimizing performance, and reducing energy consumption emissions, and is an important guarantee for promoting the progress and development of aviation technology.

For the friction force model, it can be divided into differential constraint model:

(8) $$\left\{\begin{array}{l} {\nu_{n}}^{+}=k{\nu_{n}}^{-},\\ {\nu_{f}}^{+}={\nu_{f}}^{-}-\mu (1+k){\nu_{n}}^{-} \end{array}\right.{\nu_{n}}^{+}$$

where

${\nu_{n}}^{+}$, ${\nu_{f}}^{+}$, ${\nu_{n}}^{-}$, ${\nu_{f}}^{-}$—the radial and tangential speeds of the rotor after and before the rubbing event,

0<k≤1—the speed decay coefficient,

μ—the tangential friction coefficient.

And the segmented smooth model is as follows:

(9) $$\left\{\begin{array}{l} F_{n}=k_{c}(r-r_{0})\\ F_{f}=\mu F_{n}, \end{array}\right.$$

where

$F_{n}$—the radial contact force during the rubbing event,

$F_{f}$—friction force during the rubbing event,

$k_{c}$—the contact stiffness,

μ—the friction coefficient,

r—the radial displacement of the rotor.

Therefore, in the nonlinear dynamics problem of friction of aeroengine rotor system, it is possible to establish the synchronous full-cycle friction model of the nonlinear rotor system, the reverse full-cycle friction model of the nonlinear rotor system, the full-cycle friction model of the elastic support–rigid rotor system, and the coupling system model of the machine double rotor and casing. These models fully reveal the stability, bifurcation boundary, and dynamic behavior characteristics of the system under different conditions, and provide an important theoretical basis for the design of aeroengine rotor systems [28].

For a single rotor–rolling bearing–casing coupling system, there is a friction force model:

(10) $$\left\{\begin{array}{l} P_{N}=k_{r}\cdot (e-\delta )\\ P_{T}=f\cdot P_{N} \end{array}\right.$$

where

r—the radial relative displacement of the rotor disk and the casing, and the expression is the

(11) $$r=\sqrt{{\left(x_{rp}-x_{c}\right)}^{2}+{\left(y_{rp}-y_{c}\right)}^{2}.}$$

where

e—the mass eccentricity,

δ—the clearance between the rotor disk and the stator,

$k_{r}$—the radial stiffness of the stator,

f—the friction coefficient,

$P_{T}$—the tangential components of the rubbing force,

$P_{N}$—the normal components of the rubbing force.

There is r<δ, no friction at that time, and only $P_{x}=P_{y}=0,r\geq \delta$ sometimes there is friction. The frictional force is then decomposed on the x and y axes:

(12) $$\left\{\begin{array}{l} P_{x}=k_{r}(1-\delta /r)[-(x_{rp}-x_{c})+f\cdot (y_{rp}-y_{c})]\\ P_{y}=k_{r}(1-\delta /r)[-(y_{rp}-y_{c})-f\cdot (x_{rp}-x_{c})] \end{array}\right..$$

Through the study of this touch, it is possible to understand more clearly the embodiment of force in the institution [29].

For the friction failure that will occur between the high-pressure turbine disk and the low-pressure turbine disk in the receiver constraint state, considering the energy dissipation in the process of friction, we employ the Lankarani-Nikravesh hysteresis force model to characterize the collision force $F_{n}$ between a rigid rotor disk and its casing. and the coulomb model is used to characterize the friction between the two:

(13) $$\left\{\begin{array}{l} F_{n}=k_{c}(\delta -\delta_{0}{)}^{\frac{3}{2}}[1+\frac{3(1-c_{e}^{2})\dot{\delta }}{4{\dot{\delta }}^{-}}]H(\delta -\delta_{0})\\ F_{f}=\mu F_{n} \end{array}\right.$$

where

$\dot{\delta }$—the radial velocity of the turbine disk,

${\dot{\delta }}^{-}$—the initial velocity,

$\delta_{0}$—the initial clearance,

$\delta_{1}$—the high-pressure turbine disks,

$\delta_{2}$—low-pressure turbine disks,

$c_{e}$—the coefficient of restitution for the collision,

μ—the friction coefficient,

$k_{c}$—the Hertz contact stiffness, which can be expressed as:

(14) $$k_{c}=\frac{4}{3\left(\frac{1-\nu_{p}^{2}}{E_{p}}+\frac{1-\nu_{c}^{2}}{E_{c}}\right)}(\frac{R_{c}R_{p}}{R_{c}+R_{p}}{)}^{\frac{1}{2}}.$$

where

$R_{p}$—the curvature radii of the rotor disk,

$R_{c}$—the curvature radii of the casing in the contact area,

$\nu_{p}$—the Poisson’s ratios of the rotor disk,

$\nu_{c}$—the Poisson’s ratios of the casing

$E_{p}$—the elastic moduli of the rotor disk,

$E_{c}$—the elastic moduli of the casing,

Based on the above model, it can be calculated that the frictional force in the x direction and the component in the direction can be expressed as

(15) $$\left\{\begin{array}{l} P_{x}\\ P_{y} \end{array}\right\}=\left[\begin{array}{ll} -cos\varphi & sin\varphi \\ -sin\varphi & -cos\varphi \end{array}\right]\left\{\begin{array}{l} F_{n}\\ F_{t} \end{array}\right\}.$$

Through the study of friction faults of high-pressure turbine disks and low-pressure turbine disks under the confinement state of the casing, the friction phenomenon can be fully simulated, the fault mechanism can be revealed, the system response can be predicted, the fault diagnosis can be provided with a scientific basis, and the engine design optimization can be guided to improve reliability and safety [30].

It can not only reveal the root cause and mechanism of friction faults and provide a key basis for fault warning and diagnosis, but also guide the optimal design of engine structure, prolong service life, reduce maintenance costs, and ensure the sustainable development of aviation.

## 4. Application of Vibration Friction Technology in the Field of Aero-Engines

Vibration and friction reduction technologies in aerospace engines are primarily applied in the fields of coatings, lubricant applications, bearings, and seal structures, as well as surface treatment processes. Various methods are employed to mitigate the adverse effects of vibration and friction within the engine. These methods aim to reduce the detrimental impacts arising from internal vibrations and friction, thereby enhancing the overall performance and reliability of the engine.

Pratt and Whitney’s turbofan engines, such as the PW1000G series, employ advanced diamond-like carbon (DLC) coating technology, primarily on high-friction components such as turbine disks, bearings, and seals. These coatings effectively reduce friction and wear during high-speed engine operations, significantly enhancing component durability. This coating technology has achieved remarkable results in improving fuel efficiency and reducing maintenance costs, thereby extending the engine’s lifespan. Compared to traditional engines, a fuel consumption reduction of 15% has been achieved [31,32].

Lubricant application: The Trent XWB engine is one of the most fuel-efficient commercial aero-engines, and its superior performance is due in part to the use of synthetic lubricants to provide stable lubrication at ultra-high temperatures [33]. In 2005, Russia developed a new fully synthetic 7.5 mm/s lubricant KA–7.5. The product standard is TY 38.4011103-2005, KA–7.5. The kinematic viscosity at −40 °C is only 7600 ${mm}^{-2}$/s, and the maximum working temperature can reach 200 °C [34]; however, the kinematic viscosity of C.M–4.5 at −40 °C is as high as 21,500 ${mm}^{-2}$/s, and the maximum operating temperature is only 125 °C, and hence, KA-7. The high- and low-temperature performance of five is better than that of mixed oil C.M–4.5, because KA–7.5 has excellent high- and low-temperature performance, and it can be used in all seasons, so it has been mass-produced and popularized, and its specific indicators are shown in the following Table 3:

The LEAP-X series engine is a large commercial aircraft engine developed by CFM International, a joint venture between General Electric (GE) of the United States and SNECMA (Safran Group) of France. CFM International unveiled the LEAP-X engine on 4 November 2008, and have since continued to innovate and improve it. The ceramic ball bearings used in the LEAP-X engine exhibit excellent properties such as a light weight, wear resistance, temperature tolerance (including low temperatures), corrosion resistance, and good precision retention. The structure of these bearings is illustrated in the accompanying figure (left). Experiments have demonstrated the great potential of full-ceramic ball bearings for service under extreme operating conditions [35,36]. Additionally, the LEAP engine employs self-lubricating carbon/graphite composite seals, which provide low friction and high wear resistance in high-temperature environments.

## 5. Conclusions

Vibration and friction reduction technologies in aerospace engines play a vital role in the modern aviation industry, directly impacting engine reliability, efficiency, and lifespan. Coatings, lubricants, and bearing structure innovations constitute the three core areas of current vibration and friction reduction systems. These technologies work synergistically to not only enhance the wear resistance of critical components, but also significantly reduce maintenance costs and fuel consumption.

Advancements in coating technology have provided better surface protection for the high-temperature components of engines. Advanced coating materials such as diamond-like carbon (DLC) and ceramic coatings offer exceptional wear resistance under extreme conditions, extending the lifespan of components. With continuous improvements in coating materials and processes, their application scope is expanding from turbine disks and blades to more critical parts, enabling aerospace engines to operate at higher temperatures and in harsher environments.

Lubricant technology provides stable and efficient lubrication protection for aerospace engines, especially in high-temperature and high-pressure environments. The emergence of synthetic lubricants, nano-lubricants, and solid lubricants has significantly reduced friction coefficients and enhanced sliding performance between components. With further advancements in nanotechnology, the performance of lubricants is expected to improve, ensuring that engines maintain optimal conditions during extended high-load operations. Innovations in bearing structure technology enable aerospace engines to maintain low friction and high durability at high rotational speeds. Technologies such as ceramic ball bearings and self-lubricating sealing materials are already being applied in new-generation engines. By reducing weight, vibration, friction, and improving wear resistance, these technologies significantly enhance engine efficiency and reliability.

In summary, the application of vibration and friction reduction technologies in the field of aerospace engines has not only significantly improved fuel efficiency and reliability, but also driven the overall engine design towards higher efficiency, lighter weight, and greater durability. In the future, with continuous advancements in materials science, nanotechnology, and intelligent manufacturing, vibration and friction reduction systems will play an even more central role in aerospace engine design, further enhancing overall engine performance and providing solid technical support for the sustainable development of the aviation industry.

## Figures and Tables

**Table 1:** Performance verification and comparison data.

Projects	4058 Lubricating Oil	SAEAS5780 Lubricating Oil	Foreign Oils	Index	Test Method
$\mathrm{Kinematic}\;\mathrm{viscosity}\;({mm}^{2}\cdot s^{-1})$					GB/T 265
100 °C	5.301	5.139	5.181	4.90–5.40	
40 °C	26.62	26.12	26.09	≥23.0	
−40 °C	10,902	12,535	11,377	≤13,000	
The rate of change in kinematic viscosity %	−2.6	−1.5	−0.7	±6	GB/T 1264.4
Flash point (opening) °C	283	260	266	≥246	GB/T 3536
Pour point °C	<−55		<−55	≤−55	GB/T 3535
$\mathrm{Evaporation}\;\mathrm{loss}\;\mathrm{rate}\;\left(204\;{}^{\circ}C,6.5\;h\right)\%$	3.2	8.6	4.0	≤10	GB/T 7325
NBR-H nitrile rubber (70 °C,72 h)	16.23	5.6	17.35	5~25	
BF Fluoroelastomer (204 °C,72 h)	21.87	7.6	21.12	5~25	SH/T 0436
Silastic (121 °C,96 h)	1.8	2.4	20.5	≤30	GJB 1820
$\mathrm{Change}\;\mathrm{in}\;\mathrm{acid}\;\mathrm{number}\;(mgKOH\cdot g^{-1})$	0.40	0.4	0.71	≤3.0	
$\mathrm{Sediment}\;\mathrm{mass}\;\mathrm{concentration}\;(10\;\mu m\;\mathrm{membrane})\;(mg\cdot {\left(100\;mL\right)}^{-1}$	1.9	0.7	0.8	≤26	
Steel	not	0.015	not	±0.2	
Silver	−0.06	−0.015	−0.07	±0.2	
Aluminum	not	0.023	not	±0.2	
Magnesium	not	−0.185	not	±0.2	
Copper	−0.17	−0.015	−0.05	±0.2	
$\mathrm{Kinematic}\;\mathrm{viscosity}\;\mathrm{change}\;\mathrm{rate},\;\left(40\;{}^{\circ}C\right)\%$	20.19	27.5	46.97	≤60	
$\mathrm{Change}\;\mathrm{in}\;\mathrm{acid}\;\mathrm{number}\;(mgKOH\cdot g^{-1})$	1.8	5.69	4.9	≤10	
$\mathrm{Sediment}\;\mathrm{mass}\;\mathrm{concentration}\;(10\;\mu m\;\mathrm{membrane})\;(mg\cdot {\left(100\;mL\right)}^{-1}$	0.6	0.6	0.6	≤25	
Steel	not	0.039		±0.2	
Silver	−0.04	−0.116		±0.2	
Aluminum	not	0.008		±0.2	
Titanium	not	−0.069		±0.2	
$\mathrm{Kinematic}\;\mathrm{viscosity}\;\mathrm{change}\;\mathrm{rate},\;\left(40\;{}^{\circ}C\right)\%$	−1.39	−0.47		≤5.0	
$\mathrm{Change}\;\mathrm{in}\;\mathrm{acid}\;\mathrm{number}\;(mgKOH\cdot g^{-1})$	3.1	5.69		≤6.0	GJB 1264.1
$\mathrm{The}\;\mathrm{amount}\;\mathrm{of}\;\mathrm{change}\;\mathrm{in}\;\mathrm{the}\;\mathrm{mass}\;\mathrm{of}\;\mathrm{No}.\;15\;\mathrm{steel}\;(mg\cdot cm^{-2})$	−0.18	0.47		≤4.0	
371 °C Vapor phase coking capacity/mg	82.0				SAE ARP5921

**Table 2:** Compatibility test with engine materials.

Project	4058 Lubricating Oil	SAEAS5780 Lubricating Oil	Foreign Reference Oils
TC4	not	not	not
Cr4Mo4v	0.013	0.015	0.018
16CrNiWMoVNbE	not	not	not
GH4I69,ZTA15,18Cr2Ni4WA	not	not	not
ZL114A	not	not	not
ZM6	−0.88 (pitting)	−1.22 (pitting)	−1.59 (pitting)
ZGCr15	not	not	not
QSi3.5-3-1.5,LY11,12Cr2Ni4A	not		not
ZM6 magnesium	not	not	−0.02 (pitting).
FX 4 fluoroelastomer	21.83	23.56	25.23
FX 16 fluoroelastomer	15.92	17.86	18.21
FX 4 fluoroelastomer	73.2	70.2	70.5
FX 16 fluoroelastomer	43.7	42.8	40.3
FX 4 fluoroelastomer	57.6	43.2	49.9
FX 16 fluoroelastomer	50.2	30.5	34.6
FX 4 fluoroelastomer	8	8	10
FX 16 fluoroelastomer	3	5	7
Rubber compatibility (70 °C, 168 h)			
Rubber expansion rate, %			
FX 4 fluoroelastomer	4.11	4.39	5.42
FX 16 fluoroelastomer	2.91	2.88	3.69
FX 4 fluoroelastomer	24.6	10.2	11.5
FX 16 fluoroelastomer	0.5	6.5	7.7
Elongation change at break, %			
FX 4 fluoroelastomer	0	0	0
FX 16 fluoroelastomer	0	0	9
Hardness change/degree			
FX 4 fluoroelastomer	8	7	8
FX 16 fluoroelastomer	3	3	3

**Table 3:** C.M–4.5 comparison with key indicators of lubricant sKA–7.5.

Project	KA−7.5	C,M−4.5	Test Method
$\mathrm{Viscosity}/{mm}^{-2}$/s	/	/	ΓOCT33
100 °C	≮7.50	≮6.5	
Freezing point/°C	≯−55	≯−35	ΓOCT20287
Flash point (opening), °C	≮200	≮411 (closed)	ΓOCT4333
$\mathrm{Acid}/mgKOH\cdot g^{1}$	≯0.15	≯0.07	ΓOCT5985
Ash/%	0.20		ΓOCT1461
Carbon residue/%	0.20	≯0.20	ΓOCT19932
Water-soluble acids and bases	Not	Not	ΓOCT6307
Mechanical impurities	Not	Not	ΓOCT6370
Moisture content	Not	Not	ΓOCT2477
$\mathrm{Density}\;(20\;{}^{\circ}C)/kg\cdot {cm}^{-3}$	≯1000		ΓOCT3900
